# *Toxocara Cati* as a Target for Feline IgA

**DOI:** 10.1007/s11686-026-01446-3

**Published:** 2026-11-11

**Authors:** Pia Rapp, Laura Braig, Antti Tuhkala, Antti Sukura, Anu Näreaho

**Affiliations:** 1 The Department of Veterinary Biosciences, University of Helsinki, Helsinki, Finland; 2 Fresenius University of Applied Sciences, Idstein, Germany; 3 Institute of Biotechnology, Helsinki Institute of Life Science HiLIFE, University of Helsinki, Helsinki, Finland

**Keywords:** *Toxocara Cati*, IgA, Antigen, ELISA, Immunoblot, Mass Spectrometry

## Abstract

*Toxocara cati* is a common intestinal parasite of cats globally. On the mucous membranes, the host’s immune system defends itself against​ invading pathogens, such as parasites, with immunoglobulin A (IgA) among other mechanisms. The precise role of IgA in *T. cati* infections is not yet understood.

We determined the total IgA levels from serum (*n* = 8) and feces (*n* = 10) of *T. cati* -infected cats before and after anthelmintic treatment with a commercial ELISA. These results were compared with IgA levels of nonparasitic cats (serum *n* = 8, feces *n* = 6). We also visualized the binding of feline IgA to​ adult *T. cati* worms with immunohistochemistry and analyzed the IgA-reactive antigens with Western blot. Five antigenic bands were further analyzed with mass spectrometry.

We found no consistency between total IgA levels in serum or feces and *T. cati* infection. In immunohistochemistry, the intestines, reproductive tracts, excretory canals, and muscle cell bodies of *T. cati* were the most immunoreactive parts when processed with either *Toxocara*-positive cat sera or pathogen-free cat serum. When comparing *T. cati* -infected and pathogen-free cat serum as IgA source in Western blot, most bands showed nonspecific IgA immunoreactivity independent of the host’s *Toxocara* status. In mass spectrometry, the most prevalent proteins in the selected antigenic bands were two uncharacterized proteins, intermediate filament proteins, endoplasmin, paramyosin, actins, and glutamate dehydrogenase. The combined findings of this study suggest that IgA has mainly nonspecific actions against *T. cati*.

## Introduction

*Toxocara cati* is a widespread feline nematode belonging to the Ascarididae family. The pooled, global prevalence of *T. cati* in cats was estimated to be 17% in a systematic review [1]. Cats and other felids are its definitive hosts, but it also has a range of paratenic hosts, such as rodents and birds. Cats get infected either by eating raw paratenic host meat (usually their prey), by the transmammary route from a queen to kittens, or by ingesting *T. cati* eggs with infective larvae from the environment [2]. After ingestion of a paratenic host or infected queen’s milk, *T. cati* larvae undergo mucosal migration in the intestine and reach patency in six weeks. In young cats, the ingestion of infective larvae from the environment causes the larvae to migrate through the liver and lungs into the small intestine, where they reach patency in eight weeks after infection. Somatic migration of larvae happens in older cats and when the queen gets infected late during pregnancy [3, 4]. *Toxocara* infection can cause respiratory and gastrointestinal signs in addition to dehydration and anemia in infected cats, especially kittens [4]. The female *Toxocara* nematode produces a large number of eggs that are released to the environment with feces [4–6]. *Toxocara* spp. are zoonotic, and humans can end up as their accidental hosts [7–9]. When diagnosing human toxocariosis, it is usually not distinguished whether the infection was caused by the canine roundworm *Toxocara canis* or *T. cati*. Therefore, the role of *T. cati* as a zoonotic threat has been suggested to be underestimated [5, 6, 10]. While completing its life cycle in the cat, *T. cati* has to avoid the host’s immune system.

Each immunoglobulin class has an important role in the host’s adaptive immune system. IgA acts against pathogens on the mucous membranes of the body. The production of IgA on the intestinal surfaces is induced by antigens either T-cell dependently from Peyer’s patches in the small intestine, or independently by plasma cells in the intestinal lamina propria, but it can also be induced nonspecifically without an antigen stimulus [11, 12]. T-cell-independent IgA synthesis is derived from the intestinal commensal bacteria, and it plays an important role in maintaining the intestinal microbial homeostasis [13, 14]. On the mucosa, two monomeric IgA antibodies are bound together with a J chain, forming a dimer [15]. This dimeric IgA binds with a polymeric immunoglobulin receptor (pIgR) on the enterocyte [12]. The IgA-pIgR compound is introduced to the intestinal lumen by transportation and endocytosis in the enterocyte [12]. The pIgR is cleaved, and the remaining receptor peptide, called the secretory component, stays bound to the dimeric IgA, forming together a compound called the secretory IgA (sIgA) [12]. It has a significant responsibility for discriminating pathogens, including parasites, from nutritional antigens and also from commensal and beneficial microbes, hence keeping up the vital balance between harmful invaders and crucial nutrients and microorganisms [16, 17]. In cats, IgA in serum is claimed to be mainly in the dimeric form, contrary to humans [18]. In earlier studies, mucosal IgA has been shown to have a role in the expulsion of intestinal helminths [19–23].

The IgA response of cats against nematode infections has not yet been extensively studied. In particular, the fecal IgA levels have not been broadly studied previously, even though IgA is the main antibody in local mucosal defense. One study has found that cats’ allergen-specific IgA response was stronger when they were infected with *T. cati* compared to noninfected ones [24]. In dogs with miscellaneous parasite infections, there are a few more studies published. In one article, the fecal IgA levels decreased in dogs infected with *T. canis* or *Giardia sp.* but significantly increased in dogs that were infected with *Cystoisospora canis*, a coccidian parasite [25]. Systemic IgA results have also been somewhat incoherent, since there have been reports of both increases in parasite-specific IgA during cestode infections [26] and decreases in serum total IgA in parasitized dogs [27].

The purpose of this study was to examine the total IgA response in serum and feces of both *T. cati* -infected and parasite-free cats, to identify gross differences associated with the infection. In addition, we wanted to visualize the binding sites of feline IgA on *T. cati* adult nematodes and to identify the IgA-binding *T. cati* antigens.

## Materials and Methods

We collected fecal and serum samples from *T. cati -*infected cats and non-infected negative controls. Fecal samples of cats eligible for this study were recruited from referring veterinarians and cat shelters in Finland from November 2024 to June 2025. Altogether 14 *T. cati* -positive and 10 negative cats were included in the study. The fecal samples were collected by the owner or the caretaker in the shelter and kept at 4 °C for a maximum of seven days before examination. Only individuals positive for *Toxocara* and negative for other intestinal parasites were accepted in the positive group. The fecal samples of negative control cats were confirmed to be free of intestinal parasites. The feces were examined with the quantitative Mini-FLOTAC method using zinc sulfate (specific gravity 1.35; sensitivity 10 eggs per gram [EPG] of feces) for nematode eggs and protozoan oocysts, and with IDEXX SNAP^®^*Giardia* Test Kit (IDEXX Laboratories; Westbrook, ME, USA) for *Giardia* antigens. *Toxocara*-positive cats were dewormed *per os* with fenbendazole for three consecutive days (nine cats), with praziquantel-emodepside spot-on (three cats), with imidacloprid-moxidectin spot-on (one cat), or with pyrantel-praziquantel *per os* (one cat). The anthelmintic was chosen by the owner or shelter and administered according to the manufacturer’s instructions and the cats’ weights. Fourteen days after deworming, new fecal samples were collected and examined with Mini-FLOTAC. All eligible fecal samples were frozen at -20 °C as one-gram aliquots for further use. Serum samples were collected from *Toxocara*-positive cats before and 14 days after treatment, and from *Toxocara*-negative cats, with venipuncture (*vena cephalica*) into serum tubes. After the separation of serum, the supernatant was collected in a 2-mL conical tube (Eppendorf, Hamburg, Germany) and kept at -20 °C.

Out of the 14 *T. cati* -infected cats, eight were male, three of them castrated. Two of the six female cats were spayed, and the reproductive status of one female was not known. The cats’ ages ranged from six months to five years (median 1.0); the age of the oldest cat was estimated. The age of one cat was not known nor estimated. All *Toxocara*-positive cats were domestic. The EPG values of *Toxocara*-positive cats varied from 120 to 13 400 (median 950). Altogether ten cats from volunteered owners and one local cat shelter were included as negative controls after their fecal samples were examined and confirmed to be free of intestinal parasites. Their ages ranged from 8 months to 15 years (median 8.5 years). Six of them were females, three of which were spayed. All four male cats were castrated. Six cats were domestic, two were Oriental, and two were Siberian.

The samples from the negative control cats were used in the enzyme-linked immunosorbent assay (ELISA) as nonparasitic controls. In immunohistochemistry and Western blot, commercial normal cat serum (2BScientific Limited, Oxfordshire, UK) was used as a negative control serum.

Adult *T. cati* nematodes were collected from naturally infected cats’ feces and vomitus, and from autopsies of naturally infected cats by the pathologists at the Finnish Food Authority (material transfer agreement, MTA). The nematodes were identified as *T. cati* by morphological features using a stereomicroscope (SZX9, Olympus, Tokyo, Japan). For reference, one male *T. canis* nematode was collected from the feces of a naturally infected dog. In addition, two female *T. canis* nematodes were collected from an autopsy of a dog by the veterinary pathologists in the Finnish Food Authority (MTA). The collected nematodes were rinsed with tap water and stored in 70% ethanol at 4 °C.

An animal experiment permission was granted for this study (ESAVI/45932/2021, with addition: ESAVI/38949/2024). The owners and the shelter managers gave their written consent via email to serum sampling and access to the cats’ data.

### Total IgA ELISA

Commercial feline double sandwich IgA ELISA kits (Immunology Consultants Laboratory Inc, Portland, OR, USA) were used to analyze the total IgA levels of serum and fecal samples. This kit is not validated to be used for feces, but the protocol for fecal samples was described by Peters et al. [28]. We tested the ELISA kit first with multiple parallel serum and fecal samples to verify the functionality and to establish the proper dilutions for both sample types. Exactly one gram (fresh weight) of feces was mixed in a 10-mL conical tube (Falcon, Corning Incorporated, Corning, NY, USA) with extraction buffer (0.01 M phosphate-buffered saline [PBS] and 0.5% Tween^®^ [Sigma-Aldrich, Saint Louis, MO, USA]). The tube was thoroughly shaken and vortexed to homogenize the sample. After centrifugation of the suspension at 1500 x *g* for 20 min at 5 °C, 2 mL of supernatant was mixed with 5 µL of protease-inhibitor cocktail (Epigentek Group Inc, Farmingdale, NY, USA), vortexed, and centrifuged at 10 000*g* for 10 min. The supernatant was collected for further use in ELISA.

Starting dilutions for the dilution series were determined with preceding tests. All samples were diluted with the commercial diluent available in the ELISA kit to four different concentrations (serum: 1:20 000, 1:40 000, 1:80 000, 1:160 000 and feces: 1:2500, 1:5000, 1:10 000, 1:20 000). The standards had concentrations of 0, 100, 200, and 400 ng/mL. The protocol was modified from the manufacturer’s instructions. Briefly, diluted samples and standards were pipetted into pre-designated wells, and the ELISA plate was incubated with a cover at room temperature for 30 min. After washes with wash buffer, horseradish peroxidase (HRP) conjugate was added, and the plate was kept wrapped in tin foil at room temperature for 30 min. After washes, chromogen-substrate solution (tetramethylbenzidine and hydrogen peroxide in citric acid) was pipetted into the wells. After 10 min of incubation wrapped in tin foil at room temperature, stop solution (sulfuric acid) was added. The ELISA plate was analyzed immediately at 450 nm wavelength with Byonoy 96 ELISA plate reader (Byonoy GmbH, Hamburg, Germany).

The total IgA levels in serum and feces were determined using standard curves. The median of each sample’s results was measured excluding any extreme outliers. The serum IgA levels of eight *Toxocara*-positive cats before and 14 days after treatment, and eight negative controls, were measured. The fecal IgA levels of ten positive cats before and after treatment, and six negative controls, were measured. Not all sample material was available from every participant cat, and due to laboratory errors, some results had to be discarded.

### Immunohistochemistry of Adult *T. cati*

Adult *T. cati* nematodes were placed in formalin for 24 hours at room temperature and then cut into three-millimeter sections. They were embedded in paraffin in the tissue processor (Tissue-Tek^®^ VIP^®^ 5 Jr., Sakura Finetek, Torrance, California, USA). The solidified paraffin block was cut into two-and-a-half-micrometer sections (Microm HM355S, Thermo Scientific, Waltham, Massachusetts, USA). The sections were assembled on glass slides, dried at room temperature, and left at 37°C overnight. The next day, the samples were rehydrated in the slide stainer (Autostainer XL, Leica, Wetzlar, Germany). After numbering the slides accordingly, the worm epitopes were retrieved in a PT module (PT Module, Lab Vision™, Fremont, California, USA) in citrate buffer with a pH of 6 at 99°C for 20 minutes. After cooling, the slides were assembled to coverplates (Epredia, Portsmouth, New Hampshire, USA) with 1× PBS. First, 190 µL of 3% hydrogen peroxide was pipetted into the coverplate wells to block any peroxidase activity in the tissue. After 10 minutes, the slides were rinsed twice with 1× PBS. Nonspecific antigen binding was blocked by adding 190 µL SuperBlock™ (Thermo Scientific, Waltham, Massachusetts, USA). After 20 minutes, either the pooled sera of three *Toxocara*-infected cats (with EPG values of 120, 190 and 580, and serum total IgA values of 15.3, 3.83 and 9.26 mg/mL, respectively) diluted 1:100, a commercial cat normal serum diluted 1:100, or SuperBlock™ only were pipetted in pre-designated coverplate wells, 190 µL each, and incubated for 60 minutes. After rinsing twice with 1× PBS, 190 µL of HRP-conjugated Goat anti-Feline IgA Heavy Chain Secondary Antibody (Bio-Techne, Minneapolis, USA) diluted 1:200 was added. After 30 minutes’ incubation and rinsing twice with 1× PBS, the recovered slides were stained by pipetting 3,3’-diaminobenzidine (Pierce™ DAB Substrate Kit; Thermo Fisher Scientific, Waltham, Massachusetts, USA) on the slides. After six minutes of horizontal incubation, the slides were rinsed with distilled water. The background tissue was stained with Harris hematoxylin (Sigma-Aldrich, Burlington, Massachusetts, USA) for 10 s and then rinsed with tap water. The slides were processed in ascending concentrations of ethanol and xylene; two minutes in each ethanol and four minutes in each xylene. The slides were finished with a coverslip workstation (HistoCore Chromax CV, Leica, Wetzlar, Germany) and examined with Zeiss Imager.A2 (Carl Zeiss Microscopy, Jena, Germany) using five-times magnification.

### SDS-PAGE and Western Blot

Ethanol (70%) preserved *Toxocara* nematodes were rehydrated in a series of decreasing ethanol dilutions. Each worm was opened longitudinally with a scalpel under a stereomicroscope (SZX9, Olympus, Tokyo, Japan), and the internal organs and hypodermis were scraped with a scalpel into 2-mL conical tubes (Eppendorf, Hamburg, Germany) with approximately 500 µL of lysis buffer, containing 8 M urea, 40 mM Tris base, and SDS. The organs of three *T. cati* male nematodes, four female *T. cati* nematodes, two *T. canis* female nematodes and one *T. canis* male nematode were dispensed in their own tubes by gender and species. The samples were then homogenized on ice with an ultrasound sonicator 25 times for one second and centrifuged for 15 min at 10,000 x *g* at 4 °C to eliminate all excess sediment. The supernatants were collected in 2-mL conical tubes (Eppendorf, Hamburg, Germany), and the protein concentrations were analyzed with a spectrophotometer (DS-C Cuvette, DeNovix, Wilmington, DE, USA). Twenty or 40 µg (according to the plan) of each *Toxocara* protein were mixed with Laemmli Sample Buffer (Bio-Rad Laboratories Inc., Hercules, California, USA) at a volume ratio of three parts of sample to one part of Laemmli Sample Buffer and heated at 95 °C for five minutes. The samples were then pipetted into the pre-designated wells of 8–16% Mini-PROTEAN^®^ TGX Stain-Free™ Protein Gel (Bio-Rad Laboratories Inc., Hercules, California, USA). Marker Precision Plus Protein™ All Blue (Bio-Rad Laboratories Inc., Hercules, California, USA) with molecular weight bands at 250, 150, 100, 75, 50, 37, 25, 20, 15 and 10 kDa was run along with the *Toxocara* proteins first at a voltage of 60 V for 10 min, and then 100 V for 60 min in Running Buffer (Bio-Rad Laboratories Inc., Hercules, California, USA). A part of the gel was stained for mass spectrometry sampling with Coomassie Brilliant Blue R-250 staining solution (Bio-Rad Laboratories Inc., Hercules, California, USA) for one hour at 37 °C and then destained with Coomassie Destaining Solution (methanol and acetic acid in distilled water) for 40 min at room temperature. The rest of the unstained gel was rinsed with distilled water and then incubated in Towbin buffer (Tris/Glycine Buffer [Bio-Rad Laboratories Inc., Hercules, California, USA], methanol, and distilled water) on ice for 15 min. The proteins were transferred from the gel to a polyvinylidene fluoride (PVDF; (Immobilon-P, Sigma-Aldrich, Burlington, Massachusetts, USA) membrane by a semi-dry transfer device (Trans-Blot Turbo, Bio-Rad Laboratories Inc., Hercules, California, USA) at a voltage of 15 V for 20 min. After rinsing with distilled water four times, five minutes each, and Tris-buffered saline (TBS) for five minutes, the membrane was incubated in blocking buffer (SuperBlock™, Thermo Scientific, Waltham, Massachusetts, USA) for one hour at room temperature. Then the membrane was cut into strips and incubated according to the blueprint in either 1:50 diluted pooled *T. cati* -infected cat sera (with EPG values of 120, 190 and 580, and serum total IgA values of 15.30, 3.83 and 9.26 mg/mL, respectively), commercial normal cat serum, or SuperBlock™ only overnight at 4 °C. After rinsing with TBS-Tween^®^ 20 (0.05%) (Sigma-Aldrich, Burlington, Massachusetts, USA) three times, five minutes each, the strips were incubated in 1:20,000 diluted secondary antibody (Goat anti-Feline IgA Heavy Chain Secondary Antibody [HRP], Bio-Techne Ireland Limited, Dublin, Ireland) for one hour at room temperature. After rinsing with TBS-Tween^®^ six times, five minutes each, the membrane was rinsed with TBS for five minutes and then incubated with Western chemiluminescence substrate (Clarity™, Bio-Rad Laboratories Inc., Hercules, California, USA) for five minutes. The chemiluminescence reaction was visualized and photographed with LAS-3000 Imager (Fujifilm, Tokyo, Japan).

### Mass Spectrometry

The protein bands were chosen for mass spectrometry according to their antigenic reactivity in the Western blot. The 100 kDa band in female *T. cati* was collected as it showed a positive reaction to *Toxocara*-infected cats’ sera only. Also, the 72 kDa bands from both female and male *T. cati* were chosen as potential specific antigens since they were immunoreactive in *Toxocara*-infected cats’ sera only. The 37 kDa band of *T. cati* male was immunoreactive in both *Toxocara*-positive cats’ sera and commercial normal cat serum (negative control) and hence chosen for mass spectrometry as a potential nonspecific antigen. The 22 kDa bands from both *T. cati* and *T. canis* males were chosen as possible corresponding antigenic bands for mass spectrometry. *Toxocara canis* was used as a reference organism for mass spectrometry since its proteome has already been characterized [29]. Mass spectrometry was performed in the Proteomics Unit at the University of Helsinki.

The protein bands that were selected after the Western blot were excised from the gel, put into individual 1-mL conical tubes (Eppendorf, Hamburg, Germany), and stored at -20 °C. The gel pieces were destained using 100 µL of 60% acetonitrile (Thermo-Fisher Scientific, Waltham, Massachusetts, USA) for 10 min, followed by 100 µL of HPLC water (Thermo-Fisher Scientific, Waltham, Massachusetts, USA) for 5 min at 37 °C repeatedly until all Coomassie staining was dissolved. Gel pieces were sequentially washed and dehydrated with 60% and 100% acetonitrile, followed by equilibration in 50 mM Tris buffer (pH 8.0). Reduction was done with dithiothreitol (DTT) (Thermo-Fisher Scientific, Waltham, Massachusetts, USA) at 60 °C for 30 min, after which the gel pieces were dehydrated with 100% acetonitrile. Alkylation was performed using iodoacetamide (Thermo-Fisher Scientific, Waltham, Massachusetts, USA) for 30 min at room temperature in the dark. After additional washing and dehydration steps, gel pieces were dried at room temperature. Digestion was performed by adding enzyme solution (12.5 ng/µL trypsin (Promega, Madison, Wisconsin, USA) in 50 mM Tris buffer; trypsin, pH 8.0) overnight at 37 °C with shaking at 300 rpm. Peptides were extracted by incubating the gel pieces sequentially with 50 µL of 60% acetonitrile for 10 min and 50 µL of 80% acetonitrile/0.1% trifluoroacetic acid (TFA, Thermo-Fisher Scientific, Waltham, Massachusetts, USA) for 10 min. Supernatants were collected after each extraction step, pooled, and dried using a SpeedVac concentrator prior to C18 cleanup. After digestion and drying, the peptides were resuspended in 100 µl of buffer A (0.1% TFA, 1% acetonitrile in HPLC-grade water and desalted with BioPureSPN PROTO 300 C18 Mini columns (Higgins Analytical, Mountain View, California, USA) according to the manufacturer’s instructions. After desalting, the samples were dried in a centrifuge concentrator (Concentrator Plus, Eppendorf, Hamburg, Germany). The dried peptides were reconstituted in 30 µl of buffer A and further diluted 1:10 with HPLC water containing 0.1% formic acid (Thermo-Fisher Scientific, Waltham, Massachusetts, USA). The desalted samples were loaded to Evotips (Evosep, Odense, Denmark) and analyzed using the Evosep One liquid chromatography system (Evosep, Odense, Denmark) coupled to a hybrid trapped ion mobility quadrupole TOF mass spectrometer (Bruker timsTOF Pro 2, Bruker, Billerica, Massachusetts, USA) via a CaptiveSpray nano-electrospray ion source (Bruker, Billerica, Massachusetts, USA) [30]. An 8 cm x 150 μm column with 1.5 μm C18 beads (Evosep, Odense, Denmark) was used for peptide separation with the 60 samples per day method (21 min gradient time). Mobile phases A and B were 0.1% formic acid in water and 0.1% formic acid in acetonitrile, respectively. The samples were analyzed with the DDA-PASEF-short_gradient_0.5s-cycletime method [30]. We used the Fragpipe analysis platform (version 23.1) with MSFragger (version 4.3) [31, 32] and Philosopher (version 5.1.0) [33] for peptide identification using raw (.d) files as input. Common contaminant and decoy sequences were appended to the *T. canis* reference proteome (UP000031036). For the MSFragger analysis, default settings were kept except that the precursor mass tolerance was set from − 20 to 20 ppm and the fragment mass tolerance to 20 ppm. Isotope error was set to 0/1/2. Peptide length was set from 5 to 50 base pairs, and peptide mass was set from 200 to 5000 Da. The maximum number of variable modifications per peptide was set to 3.

### Statistical Analysis

Statistical analysis was performed with IBM^®^ SPSS^®^ 31.0.2.0 for Windows^®^. A *P*-value of under 0.05 was considered statistically significant. The statistical differences between the IgA levels before and after treatment were executed using Wilcoxon Signed-rank test. The comparison between IgA levels of infected and noninfected cats was done with a nonparametric Mann-Whitney U test. The correlations between EPG values and IgA levels were analyzed with the Spearman correlation test.

## Results

### IgA ELISA

**Fig. 1:**
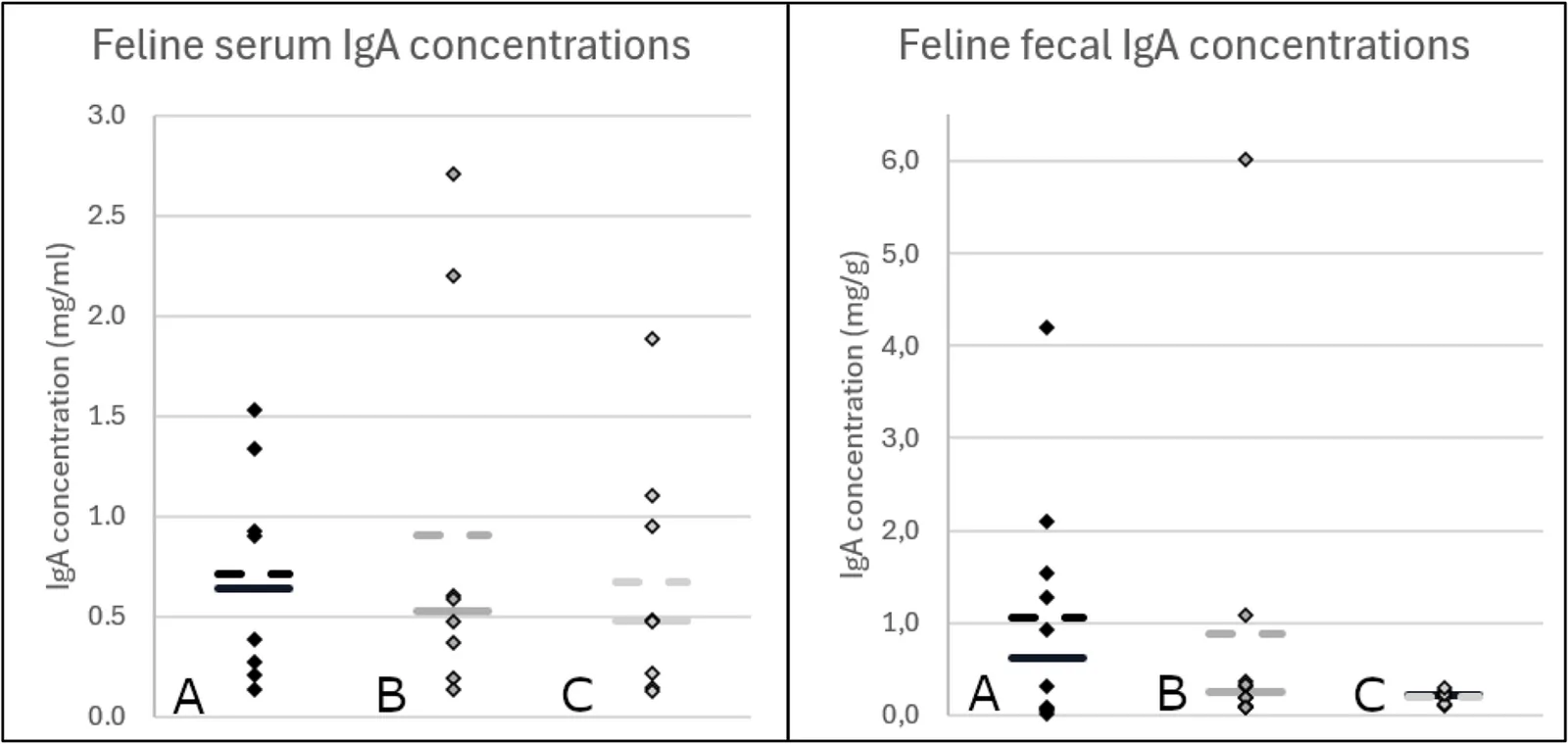
Dot plots of total serum (left) and fecal (right) IgA concentrations of *T. cati-*infected cats before treatment (**A**) and 14 days after treatment (**B**), and nonparasitic cats (**C**). Median values are marked with solid lines and mean values with dotted lines. Serum IgA was measured from eight cats, and fecal IgA from ten cats (plots A and B) and six cats (plot C)

Eight *Toxocara*-positive cats had total serum IgA levels ranging from 1.34 to 15.3 mg/mL (median 6.43 mg/mL) before treatment. After treatment with anthelmintics, serum IgA levels ranged from 1.33 to 27.1 mg/mL (median 5.28 mg/mL). The differences between the IgA serum levels in the infected cats’ group before and after treatment were not statistically significant. The serum IgA levels increased in four cats and decreased in four cats after treatment. The eight negative control cats’ serum IgA levels ranged from 1.25 to 18.8 mg/mL (median 4.78 mg/mL). There was no significant statistical difference in total IgA levels in serum between infected cats before treatment and negative control cats. When the serum IgA levels of all non-infected cats (after treatment and negative controls) were compared with IgA levels before treatment, no statistically significant difference was found. The comparison of IgA levels in serum of infected cats before and after treatment, and the negative control cats, is illustrated in Fig. 1. Individual results of *T. cati*-positive and nonparasitic cats are presented in Tables 1 and 2, respectively.

**Table 1:** Characteristics of *T. cati* -infected cats (*n* = 14) and their total fecal and serum IgA levels before and after anthelmintic treatment.

Cat no.	EPG	Age	Housing/breed	Gender	Anthelmintic	F-IgA before treatment (mg/g)	F-IgA after treatment (mg/g)	S-IgA before treatment (mg/ml)	S-IgA after treatment (mg/ml)
1	13,400	6​m	Shelter/d	Male	FBZ	1.28	1.08	1.34	1.92
2	4340	6​m	Pet/d	Male	FBZ	ND	ND	9.03	4.74
3	3690	7​m	Pet/d	Male	FBZ	ND	ND	2.70	1.33
4	3290	​8​m	Pet/d	Male/n	PRT + PRZ	0.08	0.10	13.37	6.02
5	2390	7​m	Pet/d	Female/n	FBZ	ND	ND	2.07	5.83
6	2120	1y	Pet/d	Female/n	IMD + MOX	0.93	0.32	ND	ND
7	990	8​m	Pet/d	Male	FBZ	0.31	0.18	ND	ND
8	910	1y	Shelter/d	Female	PRZ + EMD	0.06	0.36	ND	ND
9	620	3y	Pet/d	Male/n	FBZ	4.20	6.01	ND	ND
10	580	2y	Shelter/d	Female	PRZ + EMD	1.54	0.10	9.26	27.09
11	540	1y	Pet/d	Male/n	FBZ	0.08	0.20	ND	ND
12	200	ND	Shelter/d	Female/ND	FBZ	2.09	0.33	ND	ND
13	190	1y	Shelter/d	Female	FBZ	0.02	0.08	3.83	3.67
14	120	5y*	Shelter/d	Male	PRZ + EMD	ND	ND	15.30	22.04

*EPG* *T. cati* eggs per gram of feces, *M* months, *y* years. *= The age of the cat in question was evaluated by the author (PR). */d* domestic. */n* neutered. *FBZ* Fenbendazole per os for three consecutive days, *PRT + PRZ* Pyrantel + praziquantel per os, *IMD + MOX* Imidacloprid + moxidectin spot-on, *PRZ + EMD* Praziquantel + emodepside spot-on. *ND* No data

**Table 2:** Characteristics of nonparasitic control cats (*n* = 10) and their total fecal and serum IgA levels.

CAT NO.	Age	Housing/breed	Gender	F-IgA	S-IgA
15	8​m	Shelter/d	Female	0.26	1.45
16	1y	Pet/oriental	Female	0.20	18.83
17	1y	Pet/d	Female/n	0.100	ND
18	1y	Shelter/d	Female	ND	1.25
19	8y	Pet/d	Male/n	0.12	ND
20	9y	Pet/siberian	Male/n	ND	2.16
21	9y	Pet/siberian	Female/n	0.23	4.84
22	10y	Pet/d	Male/n	ND	11.05
23	13y	Pet/d	Female/n	ND	4.72
24	15y	Oriental	Male/n	0.29	9.47

*M* months, *y* years. ​*/d* domestic. */n* neutered. *ND* No data

The total fecal IgA levels of 10 *Toxocara*-positive cats before and 14 days after treatment, and six negative controls, were measured. IgA levels in feces ranged from 0.02 to 4.2 mg/g of feces (median 0.62 mg/g) before and 0.08 to 6.01 mg/g (median 0.26 mg/g) after treatment with anthelmintics. There was no statistically significant difference found in fecal IgA levels between groups before and after treatment. The IgA level in feces increased after treatment in five cats and decreased in five cats. The fecal IgA levels of negative control cats ranged from 0.10 to 0.29 mg/g (median 0.21 mg/g). The difference between *Toxocara*-infected cats’ fecal IgA levels and non-infected cats was not statistically significant. When all non-infected fecal IgA levels were compared with *Toxocara*-infected cats, the statistical difference was not significant. The fecal IgA levels of the three groups (before and after treatment, and the negative control cats) are compared in Fig. 1.

Two out of four *Toxocara*-positive cats that had both their fecal and serum IgA levels measured had an increase in the fecal IgA and simultaneously a decrease in the serum IgA 14 days after anthelmintic treatment, and vice versa in two cats. In our study material, the EPG values had a moderately negative association with serum IgA levels (Spearman Correlation Coefficient *r*=-0.571) and no association with fecal IgA levels (*r* = 0.103). Neither of these results was statistically significant.

### Immunohistochemistry of Adult *T. cati*

**Fig. 2:**
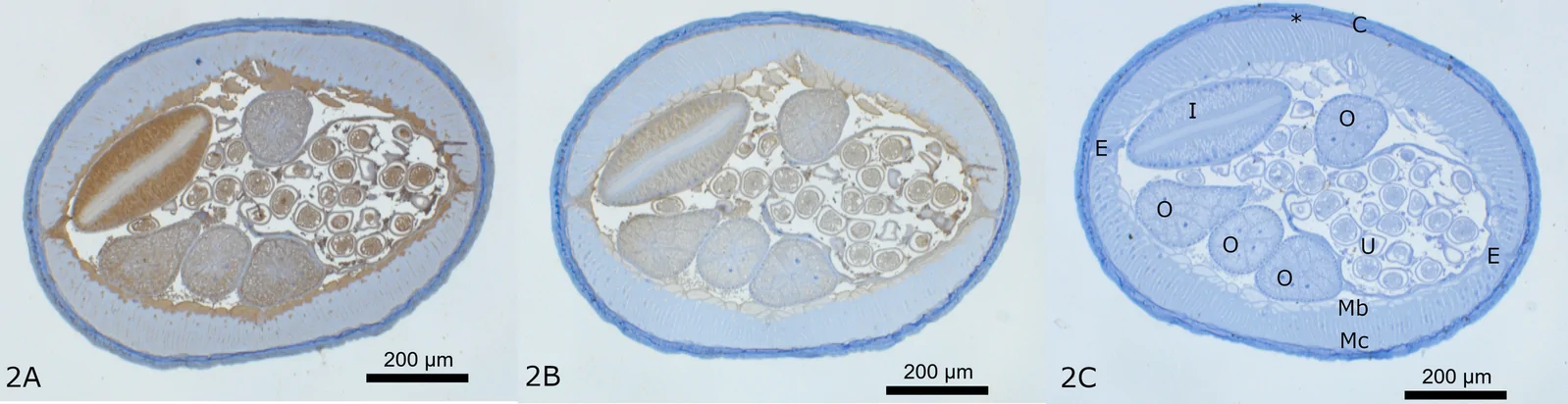
Cross-sections of a female *T. cati* adult after IgA immunohistochemistry with DAB staining. Primary antibody sources were pooled serum of *T. cati* -positive cats (**2A**) and commercial normal cat serum as a *Toxocara*-negative control (**2B**). As a non-serum control, SuperBlock^®^ was used (**2C**). I = intestine, O = ovary, U = uterus containing eggs, Mb = layer of muscle cell bodies, Mc = layer of muscle cells (contractile parts), E = excretory canal, C = cuticula. The hypodermis between the cuticula and the muscle layer is marked with an asterisk (*)

**Fig. 3:**
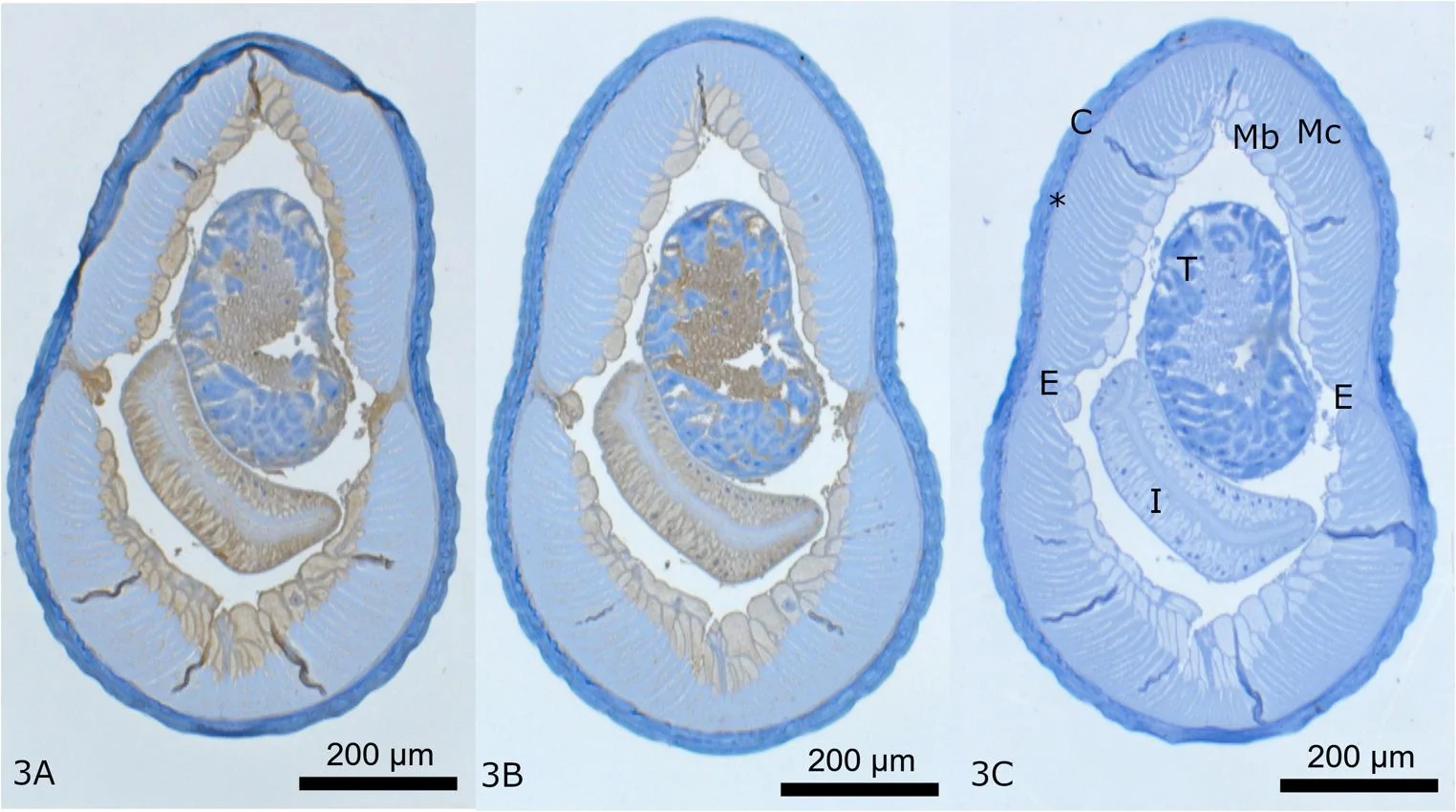
Cross-sections of a male *T. cati* adult after IgA immunohistochemistry with DAB staining. Primary antibody sources were pooled serum of *T. cati* -positive cats (**3A**) and commercial normal cat serum (**3B**). As a control, SuperBlock^®^ was used (**3C**). I = intestine, T = testis containing sperm, Mb = layer of muscle cell bodies, Mc = layer of muscle cells (contractile parts), E = excretory canal, C = cuticula. The hypodermis between the cuticula and the muscle layer is marked with an asterisk (*)

Immunohistochemistry of *T. cati* nematodes showed IgA binding especially in the intestine, uterus, eggs, sperm, the layer of muscle cell bodies, hypodermis, and excretory canals (Figs. 2 and 3). The immunoreactivity was weaker with nematode sections incubated in normal cat serum (negative control), compared to *Toxocara*-positive serum; however, nonspecific staining was evident. Cross-sections incubated in SuperBlock™ only showed no immunoreactivity to IgA.

### SDS-PAGE and Western Blot

**Fig. 4:**
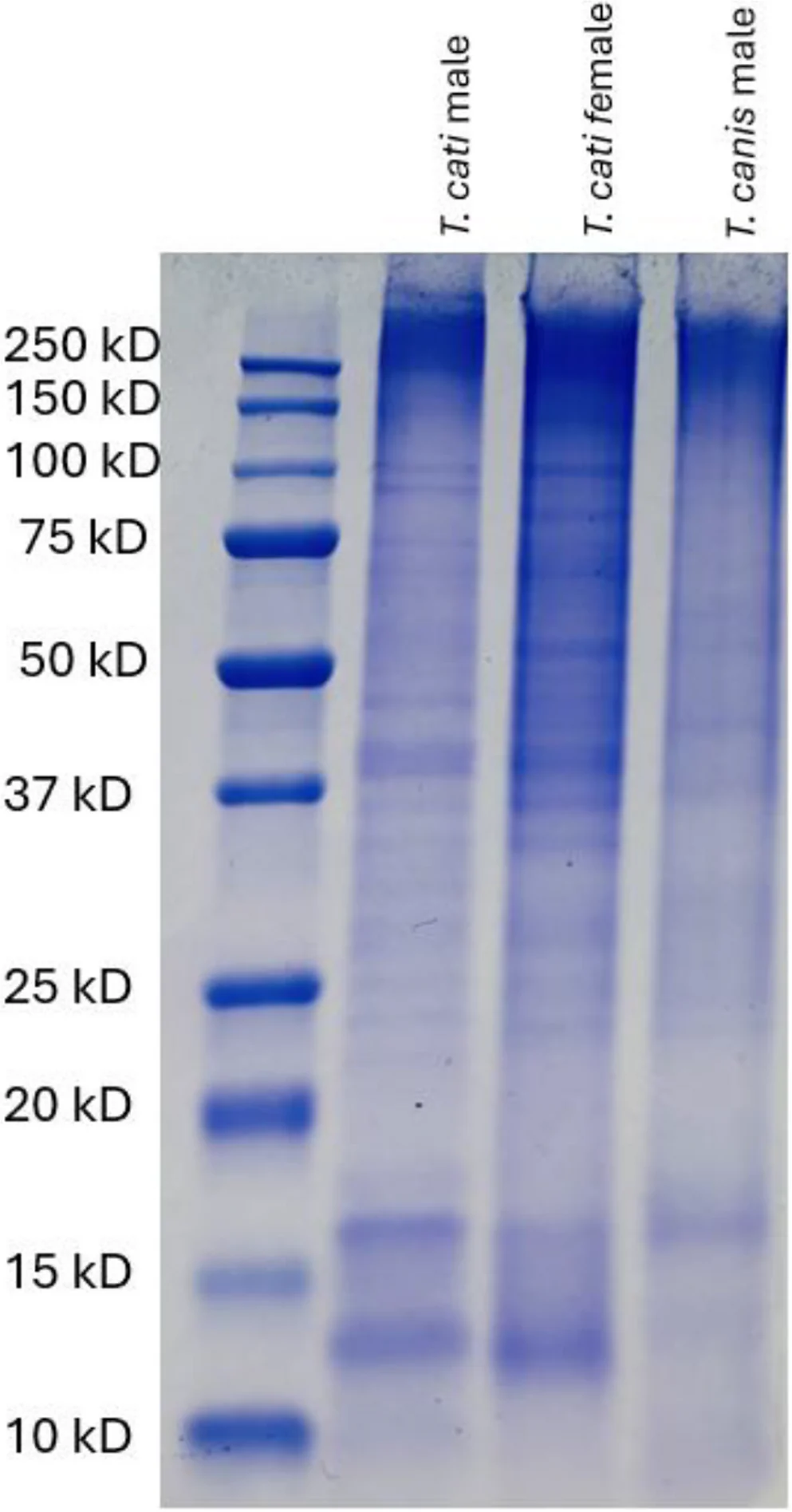
SDS-PAGE gel of *T. cati* male and female, and *T. canis* male (reference) worm antigens (20 µg each) with Bio-Rad Precision Plus Protein™ All Blue marker. The protein bands are visualized with Coomassie Brilliant Blue staining

The SDS-PAGE gel of *T. cati* male and female worms and *T. canis* male worm is shown in Fig. 4. The most prominent *T. cati* proteins were at 100, 90, 48, 40, 38, 17, and 13 kDa in male and 100, 80, 51, 38, 36, 35, 17 and 12 kDa in female *T. cati*. *Toxocara canis* male antigen was used here to compare the two species.

**Fig. 5:**
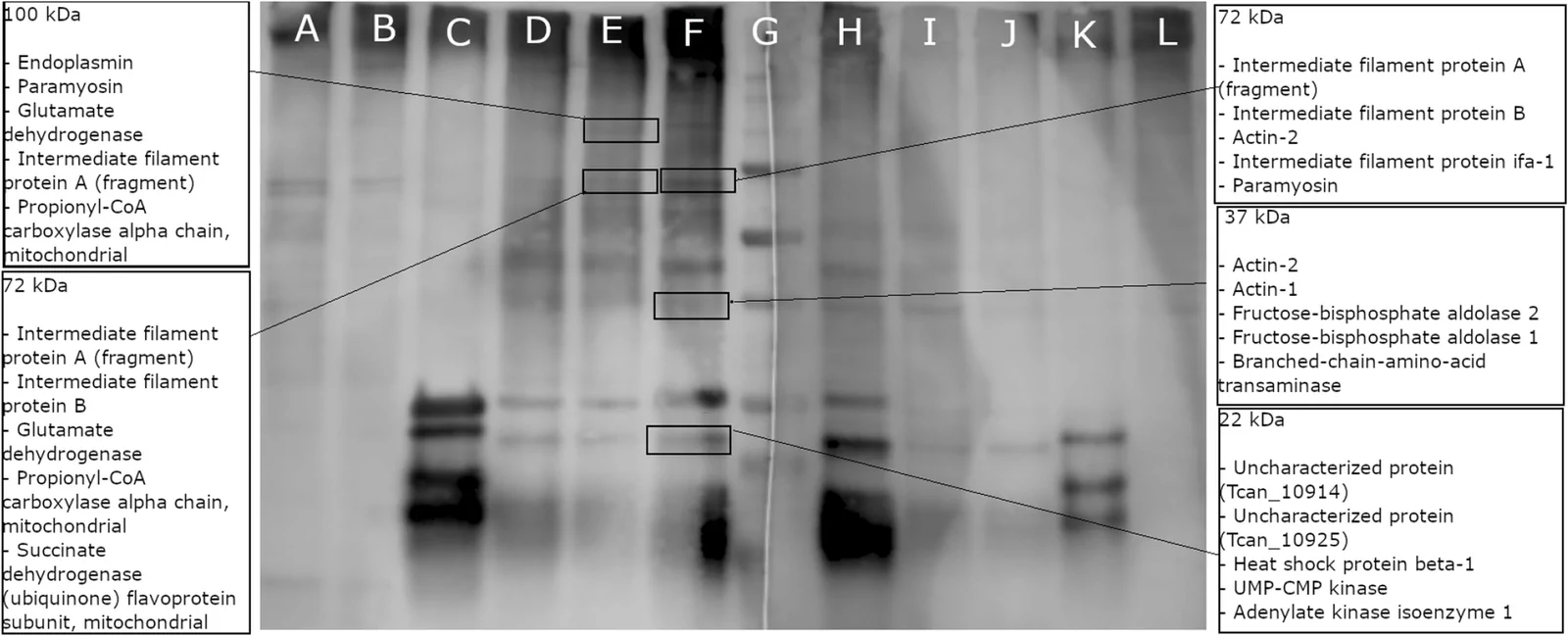
Western blot of *T. cati* and *T. canis* (reference) showing bands reactive to feline IgA. **A** = *T. canis* female (40 µg); **B** = *T. canis* female (20 µg); **C** = *T. canis* male (20 µg); **D** = *T. cati* female (40 µg); **E** = *T. cati* female (20 µg); **F** = *T. cati* male (20 µg); **G** = marker; **H** = *T. cati* male (20 µg); **I** = *T. cati* female (20 µg); **J** = *T. cati* female (40 µg); **K** = *T. canis* male (20 µg); **L** = *T. canis* female (40 µg). Lanes A to F were incubated in *T. cati* -positive feline sera, and lanes H to L were incubated in commercial normal cat serum (negative control). Bands that were analyzed with mass spectrometry are marked with a rectangle. The five most abundant proteins from each band are listed in equivalent boxes

In the Western blot (Fig. 5), we recognized IgA immunoreactive *T. cati* antigens at 250, 150, 105, 72, 70, 60, 44, 37, 25, 22, 18 and 16 kDa in male and 250, 100, 72, 70, 60, 44, 37, 25, 22, 18 and 16 kDa in female *T. cati*. Out of these antigens, the ones at 44, 37, 25, 22, 18 and 16 kDa in both genders were also IgA immunoreactive in normal cat serum (negative control).

### Mass Spectrometry

Altogether 426 *T. cati* proteins (with a protein probability of 0.9 or more) were detected from the examined five antigenic bands. Twenty-eight proteins out of the detected proteins were uncharacterized. The five most abundant proteins in each Western blot band analyzed are shown in Fig. 5.

## Discussion

We compared the IgA levels in serum and feces of *T. cati* -infected and nonparasitic cats to assess if the infection has a large-scale impact on the total amount of IgA. In serum samples, no trend in total IgA values before infection and after treatment could be seen. The fecal total IgA levels of *T. cati-*infected cats were higher before anthelmintic treatment than after treatment and compared to nonparasitic cats; however, the differences were not statistically significant between the groups. There are several known factors influencing individual feline IgA levels, such as age, dietary fiber content, subclinical viral or immune-mediated disease [34–37]. Senior cats tend to have higher serum IgA levels than younger adults [35], but similar studies of fecal IgA are lacking. In our study material, this difference was not observed in either serum or feces. However, in this study the sample size was too small to observe any associations between age and IgA levels. Other parasitic infections could also affect IgA levels, although we ruled out the most important intestinal parasites with flotation and *Giardia*-antigen testing. In *Toxoplasma gondii* infection, for example, the serum IgA levels have been shown to increase in the cat after the oocyst shedding period [38]. Retinoic acid (vitamin A) is an important inducer of switching B cells into IgA-producing plasma cells [39]. Therefore, it must be taken into consideration that the lack of dietary retinoic acid due to, for example, starvation in feral cats could reduce the IgA levels secreted from B cells on the mucosa [12]. In our study, six cats were recently caught by the local animal shelter with a possible lack of a balanced diet before arrival. Stress has also been shown to affect IgA response in different species, and the results have been somewhat controversial [40–43]. It seems that acute stress tends to cause an increase in IgA levels, but the IgA level decreases when the stress continues [41, 44]. The production of IgA is also stimulated by intestinal bacteria [14], which can explain individual differences between cats, especially considering that age-dependent changes in feline microbiota have also been detected earlier [45]. In earlier studies done with dogs, anthelmintic treatment has also been shown to have some effect on intestinal microbiota [46, 47]. To our knowledge, no similar studies have yet been published on cats, but one study on Amur tigers reached a similar conclusion [48]. In a systematic review covering 25 research articles about ruminants, rodents and pigs, the IgA response towards intestinal helminths was regarded as being diminishing rather than constraining [49]. In the review, both serum and fecal IgA were found to be higher in animals that were more resistant to intestinal helminths [49].

In humans with *Toxocara* infection, serum IgA has been especially valuable when following response to therapy, but not so much in the initial diagnostics [50]. However, the accidental nature of human *Toxocara* infection is totally different from that of definitive hosts. With cats, neither serum nor fecal total IgA seems to have any value in either; the changes in IgA levels were not coherent in serum and feces of individual cats. The cats involved in this study were already in the patent phase of the infection, which means that the cats had already been infected for weeks before the samples were taken. Based on these results, we cannot say if the phase of the infection would have a difference in results.

For total fecal IgA, we used a commercial ELISA kit that has been used earlier for analyzing feline fecal IgA, even though it is designed for serum and plasma samples [51]. For serum and plasma, there are several ELISA kit options, but feces is a more complex sample material, and there were no kits commercially available specifically designed for feline fecal IgA. The absolute fecal IgA concentrations cannot thereby be verified in this study, but the relations between samples can be compared. Since we wanted to use naturally infected cats as material and not perform experimental infection, we could not standardize the ages or other background factors, and the sample size remained small. We can provide an initial overview of the relations between feline total IgA and *T. cati* infection, but the subject needs broader experiments in the future.

For this study, we used serum as the source of IgA for immunohistochemistry and immunoblot, as it has been shown to also reflect the mucosal IgA well in dogs whose serum IgA is mostly in dimeric form, as it is in cats [52, 53]. In addition, the serum of pathogen-free cats was available commercially, contrary to feces.

The immunohistochemistry suggests that IgA-immunoreactive *T. cati* antigens are found in the intestines, muscles, hypodermis, sperm, uterus, eggs, and excretory canals of *T. cati*, but the staining was non-specific. The finding is supported in the proteomic analysis: there are IgA-immunoreactive proteins in *T. cati* that are not specific to this parasite only but are also found in other organisms.

We used SDS-PAGE to determine the protein profile of *T. cati*. In a study by Soleyman et al. [54], where *T. cati* protein bands were run in 2D-electrophoresis, the most prominent bands were found to be approximately 35, 70, and 140 kDa, which partly coincide with our results. In the Western blot, we were able to recognize some specific IgA immunoreactive *T. cati* antigens (Fig. 5), but nonspecific IgA immunoreaction was evident, which is consistent with our nonspecific immunohistochemistry findings. The worm cuticle was left out of the proteomics because it is relatively inert with respect to antibody-antigen interactions [55], which we also proved in immunohistochemistry (Figs. 2 and 3).

Some proteins identified in this study have been studied in other parasites earlier. Fructose-bisphosphate aldolase and galectin have earlier been described as IgA reactive in the ovine nematode *Teladorsagia circumcincta* [56]. In addition, enolase, actins and fructose-bisphosphate aldolase of trematode *Schistosoma haematobium* have been identified as human IgA-reactive earlier [57]. Paramyosin, a protein derived from muscle tissue, has earlier been recognized as IgA-reactive in ovine *T. circumcincta*, and has been suggested to be a possible target for helminth vaccines [58–63]. Endoplasmin, a heat shock protein also known as gp96, that has also been identified in the trematode *Schistosoma* spp. proteome, is an inflammatory mediator that takes part in activating T cells by stimulating macrophages and dendritic cells, and in phagocytosis [64]. Propionyl-CoA carboxylases take part in the worm’s energy metabolism [65]. Regarding other antibodies, out of the proteins detected in this study, actins of schistosomes and another trematode *Fasciola hepatica* have been identified earlier to be immunoreactive with human IgG, and suggested to be potential targets of trematode drugs or tools for trematode vaccines [66]. Enolase from nematode *Brugia malayi* has been shown to be reactive with human IgM [67]. In addition, paramyosin and heat shock proteins of cestode *Echinococcus multilocularis* have been found reactive to mouse IgG previously [68]. Interestingly, paramyosin from nematode *Trichinella spiralis* has also been found to inhibit the activation of complement in humans, and similar findings of paramyosin from trematode *Schistosoma mansoni* and cestode *Taenia solium* acting against complement activation have also been observed in guinea pigs [69, 70].

Our finding suggests that the IgA-related defense is directed to the same antigens in many organisms, rather than specifically against each pathogen. This must be beneficial to the host. However, we only identified some of the antigens here, and to better understand the role of IgA in parasitic infections, these proteins, among other antigens of *T. cati*, should be studied further.

## Conclusions

We conclude that feline IgA mainly binds to *T. cati* antigens that are common in other organisms as well. This nonspecific binding may help IgA to defend faster against *Toxocara* as well as other invaders in the gut. No statistically significant associations between total IgA, in serum or feces, and Toxocara infection status were found in this study, probably due to the same reason: IgA-reactive antigens are commonly found in many organisms. However, some antigens in *T. cati* adults are unidentified in our study, and their meaning remains unresolved. The small sample size limits the power of this study to detect possible correlations between total IgA levels and *T. cati* infections.

## Data Availability

Study data are available upon request via email from the author (PR).

## Declarations

### Artificial Intelligence (AI)

Writing assistant software Grammarly (free version) was used to improve the grammar and spelling of this text. ChatGPT (OpenAI, free version) was used as a guide for the statistical analysis, but no data were ever handled by AI in any way. No analyses or content were generated with AI. All advice given by AI was reviewed and assessed before use, and the corresponding author takes full responsibility for all the contents of this article.
